# Human Blood Exosomes: Isolation and Characterization Methods, Variability, and the Need for Standardized Protocols—A Review

**DOI:** 10.3390/biomedicines13122970

**Published:** 2025-12-03

**Authors:** Elena Sánchez-Vizcaíno Mengual, Laura Cordero, Hernán Pinto

**Affiliations:** Meta Cell Technology, 08173 Sant Cugat, Spain; lcordero@clinipro.net (L.C.); hpinto@clinipro.net (H.P.)

**Keywords:** exosomes, extracellular vesicles, quantification, isolation method, platelet-rich plasma (PRP), photothermal biomodulation

## Abstract

**Background/Objectives**: As bioactive extracellular vesicles, exosomes participate in cellular communication and disease mechanisms, yet their structural complexity continues to challenge standard analytical methodologies. This review summarizes published studies reporting exosome concentrations in human plasma, serum, and platelet-rich plasma from healthy individuals and highlights methodological differences. **Methods**: A comprehensive PubMed search (1986–31 August 2025) was performed using terms related to exosomes and their quantification, excluding cancer- and disease-related studies. Eligible articles reported exosome concentrations in plasma, serum, or platelet-rich plasma using particle-counting techniques such as nanoparticle tracking analysis, flow cytometry, or tunable resistive pulse sensing. **Results**: Twenty-two articles, including 167 healthy donors, met the inclusion criteria. The following mean concentration ranges were reported: plasma (n = 18), ranged from 4.50 × 10^8^ to 6.70 × 10^11^ particles/mL with differences by quantification method; serum (n = 10), from 5.30 × 10^8^ to 2.13 × 10^11^ particles/mL; non-activated platelet-rich plasma (n = 1), 7.52 × 10^9^ particles/mL; activated platelet-rich plasma (n = 3), 4.87 × 10^10^ to 7.16 × 10^10^ particles/mL; and preconditioned platelet-rich plasma with photothermal biomodulation (n = 2), 2.53 × 10^11^ to 2.99 × 10^11^ particles/mL. **Conclusions**: Isolation and quantification methods exhibit high variability, which strongly influences the overall quantity and quality of the exosomes obtained. Characteristics, including cargo composition, purity, and exosome integrity, must be considered when developing validated methods. Furthermore, emerging evidence suggests that PTBM preconditioning can increase exosome release from cells. In summary, rigorous standardization of protocols is essential to advance the scientific understanding and the clinical potential of exosome-based therapies.

## 1. Introduction

Extracellular vesicles (EVs) are spherical phospholipid bilayers ranging in size from 30 to 2000 nm [1]. Nearly all cell types secrete EVs, which are found in most biological fluids, including blood, urine, saliva, breast milk, bronchial lavage, cerebrospinal fluid, and amniotic fluid [2]. EVs can be categorized into exosomes, microvesicles (MVs), and apoptotic bodies based on size, origin, composition, and function [3]. Exosomes participate in both normal biological functions and disease, directly contributing to processes such as cell communication [4], immune response [5], and tumor dissemination [1].

Exosomes contain bioactive substances, including proteins, lipids, and genetic information, such as mRNA and non-coding ribonucleic acids (RNAs), including microRNAs (miRNAs), and their outer layers display additional proteins [6]. The process of biogenesis and exosome delivery occurs when multivesicular bodies fuse with the plasma membrane [7]. Nevertheless, the molecular mechanism underlying this process remains unclear [8]. Exosome release is influenced by several physical and chemical factors, as well as cellular conditions, including lipopolysaccharide, tumor necrosis factor-alpha, interferon-gamma, low oxygen levels, calcium, exposure to chemotherapy drugs, temperature, and oxidative stress [9].

### 1.1. Exosome Clinical Applications

Due to their biocompatibility, low immunogenicity, and ability to cross the blood–brain barrier [10], exosomes have garnered significant interest for clinical applications [11,12]. One of the principal areas of research is for regenerative medicine, where promising outcomes have been reported in tissue repair and regeneration [13], including in cancer [14], orthopedics [15], lung regenerative medicine [16], ophthalmology [17], hair regeneration [18], and brain disorders [19]. Exosomes also hold potential for diagnosis and prognosis in cancer [20], cardiovascular [21], neurological [22], autoimmune [23], and infectious diseases [24], through minimally invasive procedures [25]. Moreover, their ability to deliver specific biomarker cargo and modulate immune responses makes them potential therapeutic agents [10,26]. Exosomes can deliver targeted therapeutics to specific cells or tissues, overcoming the limitations of traditional delivery methods [27]. For instance, they are being explored for the development of exosome-based vaccines [28].

Despite these advances, several challenges remain. Key issues include optimizing isolation and purification methods, as well as concerns about the stability and bioavailability of exosomal cargo [11,12]. In addition, the regulatory environment related to exosome-based diagnostics and therapeutics is still evolving [29].

Given these considerations, accurate detection and quantification are essential. However, their complexity in size and structure poses a challenge even for gold-standard methodologies. Variability in isolation and RNA purification techniques often results in inconsistent data, hindering the comparability of findings across studies [2].

### 1.2. Exosome Isolation Methods

The current methods for exosome isolation include ultracentrifugation (UC), ultrafiltration (UF), precipitation, immunoaffinity-based capture (IAC), and microfluidic techniques.

**Ultracentrifugation (UC):** UC separates exosomes from biological samples based on size and density through sequential centrifugation. Initial low-speed spins remove cells and debris, followed by ultracentrifugation (>100,000× *g*) to pellet exosomes for further analysis [30].

**Ultrafiltration (UF):** UF uses membranes with defined pore sizes to isolate exosomes based on size. Typically, larger pores remove debris, while smaller ones (50–200 nm) retain exosomes and exclude smaller molecules [31].

**Precipitation:** This technique uses polymers, such as polyethylene glycol (PEG), to reduce exosome solubility, thereby inducing aggregation. After incubation at 4 °C, exosomes are collected by low-speed centrifugation [32].

**Immunoaffinity-based capture (IAC):** IAC uses antibodies targeting specific exosomal surface proteins, enabling selective, high-purity isolation of cell type-specific subsets, including tumor-derived exosomes [30].

**Microfluidic techniques:** These methods isolate exosomes based on size, affinity, or a combination of both. Size-based isolation includes techniques such as microfiltration, which uses micropores to separate larger particles from exosomes; size-exclusion chromatography (SEC), which separates exosomes by passing them through a microfluidic channel; and viscoelasticity, which utilizes the elastic properties of a fluid to separate particles, pushing larger debris away from exosomes [33].

### 1.3. Exosome Quantification Methods

The primary methods for a rapid and efficient quantification of exosomes include nanoparticle tracking analysis (NTA), flow cytometry (FCM), tunable resistive pulse sensing (TRPS), electron microscopy, dynamic light scattering (DLS), microfluidics-based detection, surface plasmon resonance (SPR), and the single-particle interferometric reflectance imaging sensor (SP-IRIS).

**Nanoparticle tracking analysis (NTA):** NTA quantifies exosomes by tracking their Brownian motion in a laser-illuminated liquid sample. Scattered light is recorded, and software calculates particle size and concentration based on individual trajectories [34].

**Flow cytometry (FCM):** FCM analyzes fluorescent-labeled exosomes and microvesicles using specialized flow cytometers, enabling multiparametric detection and quantification [35].

**Tunable resistive pulse sensing (TRPS):** TRPS measures changes in electrical resistance as individual exosomes pass through a nanopore, enabling determination of particle size, concentration, and charge [36].

**Electron microscopy (EM):** EM, especially transmission electron microscopy (TEM), provides a high-resolution image to count individual vesicles and assess their morphology. TEM confirms sample purity and structural integrity. To improve its effectiveness, it is often used in conjunction with other techniques, such as NTA or FCM, which provide population-level quantification [36].

**Dynamic light scattering (DLS):** DLS quantifies exosomes by illuminating a sample with a laser and measuring fluctuations in scattered light resulting from the Brownian motion of the vesicles [37].

**Microfluidics-based detection**: Microfluidic devices isolate and analyze exosomes from small liquid samples using integrated on-chip assays. Separation can be performed using SEC, immunoaffinity, or dielectrophoresis, enabling the selective detection of 30–150 nm vesicles [38].

**Surface plasmon resonance (SPR):** SPR quantifies exosomes by detecting changes in the refractive index when exosomes bind immobilized antibodies [39].

**Single-particle interferometric reflectance imaging sensor (SP-IRIS):** SP-IRIS quantifies exosomes using a specialized chip with functionalized antibodies to capture individual vesicles from a fluid sample [40].

When selecting an exosome isolation or quantification method, it is essential to consider cost, time required, and the specific advantages and disadvantages of each approach [41]. For example, some methods may include particles in their counts that are not exclusively exosomes, which could affect the accuracy of the results [11]. Some shortcomings of these techniques are outlined in Table 1 [11,32,37,42,43,44,45,46,47,48,49].

Aside from the natural biogenesis of exosomes, different methods have been studied to alter their production. These methods are based on electrical stimuli, pharmacological agents, electromagnetic waves, sound waves, shear stress, cell starvation, alcohol, pH, heat, and genetic manipulation [54,55]. However, many of these strategies may alter exosome properties and functionalities, and the exact mechanisms responsible for this increase are generally unknown. Improving exosome production and biological function is critical for promoting their clinical applications.

Given the variability in reported exosome concentrations across studies in human plasma, serum, and platelet-rich plasma from healthy individuals, this review aims to summarize available data and highlight methodological differences that underscore the need for standardized operating procedures (SOPs) and for accurate protocols.

## 2. Materials and Methods

The literature search was conducted in the PubMed database, covering studies from 1986, when the first article on exosomes in humans was published, to 31 August 2025 (Table 2). Search queries included keywords such as “exosomes” or “extracellular vesicles,” combined with terms like “particles” or “particles/mL” (to identify quantification data), along with “concentration” or “quantification” and sample type descriptors (“plasma,” including platelet-rich plasma, or “serum”). To focus on healthy donors, records associated with terms such as “cancer” or “metastasis” were excluded. Terms were adjusted according to the terminology for this search type, and articles on cancer, diseases, or pregnancy without data from healthy volunteers were excluded. The complete list of search strings and translation into PubMed format is provided in Supplemental Table S1.

After obtaining the search results by term, they were crossed with the search for “exosomes or extracellular vesicles.” Following this search, the database was manually cleaned to remove studies not aligned with the study’s objective. Inclusion criteria were limited to data from human samples from healthy donors with exosome quantification in blood plasma, serum, or PRP, expressed as particles/mL. Following the compilation of articles obtained through the systematic literature review, an additional set of publications was gathered via a targeted Google search to capture relevant works that had not appeared in the initial search.

## 3. Results

**Figure 1:**
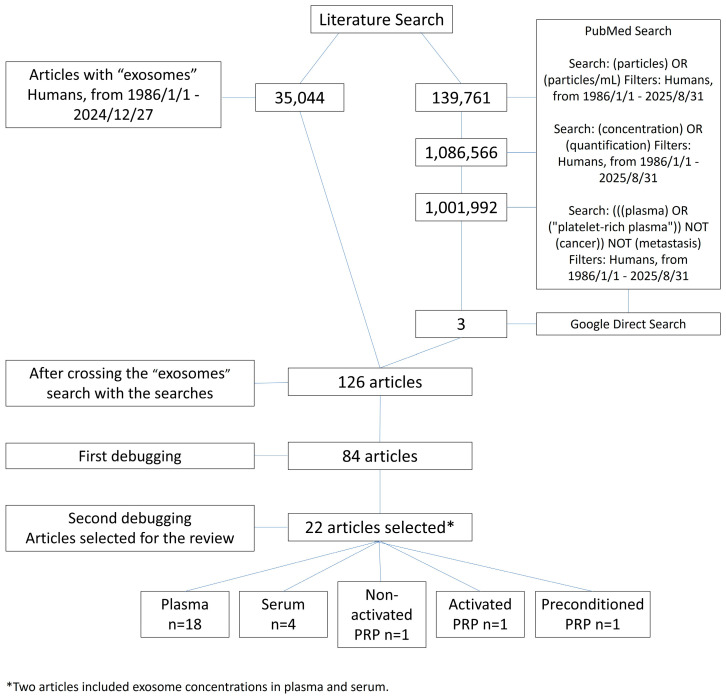
Flow diagram of the literature review process used to identify clinical research papers reporting exosome concentrations in plasma, serum, non-activated PRP, activated PRP, and preconditioned PRP.

The search for “exosomes” or “extracellular vesicles” yielded 35,044 articles. The details of the search are listed in Supplemental Table S1. After crossing the search results of the terms “serum”, “plasma”, or “platelet-rich plasma”, “concentration”, or “quantification”, and “particles” or “particles/mL” with the search for “exosomes” or “extracellular vesicles”, a total of 126 articles were obtained. In the direct Google search, three articles were included. After removing articles with out-of-scope titles, 84 articles were selected for final review. After the second screening, in which the main text was analyzed in-depth, 22 articles were selected, involving around 167 healthy donors with 29 exosome quantifications in plasma from 18 articles [56,57,58,59,60,61,62,63,64,65,66,67,68,69,70,71,72,73], 10 in serum from 4 articles [60,62,74,75], 1 in non-activated PRP from 1 article [76], 3 in PRP activated with different activators (calcium gluconate, thrombin, and thrombin and calcium gluconate) from 1 article [76], and 2 in preconditioned PRP (blue light 467 nm, 1.0 J/cm^2^, 37 °C; and blue light 467 nm, 2.0 J/cm^2^, 37 °C) [77] (Figure 1).

The mean exosome concentration in plasma ranged from 4.50 × 10^8^ to 6.70 × 10^11^ particles/mL. Stratified by quantification method, the mean concentration ranged from 3.50 × 10^6^ to 1.50 × 10^10^ particles/mL for FCM, from 4.50 × 10^8^ to 6.70 × 10^11^ particles/mL for NTA, and from 9.05 × 10^8^ to 4.36 × 10^10^ particles/mL for TRPS. In serum, the mean exosome concentration, assessed by NTA, ranged from 5.30 × 10^8^ to 2.13 × 10^11^ particles/mL. The nanoflow analysis exosome mean concentration was 7.52 × 10^9^ particles/mL for non-activated PRP. For activated PRP samples (including all activation methods—calcium gluconate, thrombin, and thrombin plus calcium gluconate), the exosome mean value ranged from 4.87 × 10^10^ to 7.16 × 10^10^ particles/mL, and for preconditioned PRP, it ranged from 2.53 × 10^11^ to 2.99 × 10^11^ particles/mL; all were assessed by NTA. Detailed measurement data and study-specific values are provided in Table 3 (plasma), Table 4 (serum), and Table 5 (PRP: activated, non-activated, and preconditioned).

## 4. Discussion

Exosome analysis remains challenging [78]. Although concentration is often emphasized, it is not a reliable indicator of exosome quality or biological relevance. Other parameters, such as sample purity, exosome integrity, and the functional significance of the cargo, are likely more critical [79] and vary widely across sample types and methodological approaches. This methodological variability complicates comparisons across studies and underscores the need for SOPs that define not only isolation and quantification strategies but also criteria for assessing exosome quality, ensuring reproducible and meaningful results to support future clinical applications.

Determining the optimal dose of therapeutic EVs is essential to maximize therapeutic effects and support their successful clinical application. Accordingly, dose-definition strategies for therapeutic EVs must consider not only vesicle concentration but also the preparation’s qualitative attributes. Sample parameters to consider include the isolation technique, viscosity, temperature, and the time of day the sample was collected, among others. Moreover, ensuring consistent physiological production and proper storage of exosomes will be critical for future clinical applications, particularly when patient-derived exosome quality or quantity may decline with disease progression.

Existing exosome isolation and quantification methods have advantages, such as high purity (SEC), rapid processing (polymer precipitation, microfluidic techniques), sensitivity (DLS, SPR), and direct counting (NTA, FCM), but also suffer from technical limitations (Table 1) or high cost (IAC, SPR). These limitations may lead to under- or overestimation of counts, or even to accurate counts with exosomes of compromised functional integrity [11]. For instance, in plasma samples, NTA often reports higher counts than TRPS or FCM [80]. Methods such as NTA and FCM count particles, while DLS measures light-scattering intensity. Thus, caution is required when interpreting reports on exosome quantification presented in scientific research or by commercial brands. The ideal standardized isolation method for exosome research and production should be easy and rapid, with high throughput, high purity, high recovery rates, and low procedural cost [30].

One alternative to minimizing quantification variation across studies is to combine different methods to leverage their strengths and mitigate their weaknesses [81]. For example, combining UC with SEC to improve results [82,83]. The combination of various methods can effectively reduce the presence of nanoscale contaminants, thereby improving purity without altering exosomes’ natural properties. Other researchers have proposed new strategies, such as dichotomic size-exclusion chromatography, for exosome isolation, achieving the best performance with higher isolation yields [84]. Exosomes can also be isolated by CD9-HPLC-IAC [85] and untouched isolation [86]. Nevertheless, purity, yield, and recovery remain ongoing challenges, and protocols should preserve exosomes’ native physicochemical environment.

Beyond methodological optimization, exosome production can be enhanced prior to isolation through cell activation or preconditioning techniques, such as photobiomodulation (PBM) and photothermal biomodulation (PTBM). A study on mesenchymal stem cell-derived exosomes (MSC-Exo) confirmed that blue light enhances angiogenesis, likely through modulating specific miRNAs [87]. Regarding preconditioned PRP samples subjected to PTBM, exposure to visible light in the 455–470 nm range (blue light) effectively increases exosome secretion rates [87], aligned with the data obtained from PTBM PRP samples, which exhibited the highest exosome concentrations, particularly compared with activated PRP, the most similar product. This observation is supported by several studies demonstrating that PBM enhances exosome secretion from dermal papillae [88], promotes endothelial cell regeneration [89], and significantly increases EV yield [90]. Cordero et al. reported that a lower fluence (1.0 J/cm^2^) significantly increased exosome counts [77]. These interventions highlight the potential to maximize yield while preserving vesicle characteristics, although further studies are needed to determine optimal sample types, treatment conditions, and quality control metrics.

Several limitations must be considered when interpreting these findings. First, there are currently no established reference values for exosome concentrations in healthy human plasma, serum, or PRP. Second, the included studies exhibited high heterogeneity in isolation and quantification techniques and sample processing. Moreover, even when the same method, such as ultracentrifugation, is used, the specific protocols differ. This variability complicates direct comparison across studies and may partly explain the wide range of reported exosome counts. Third, the relatively small number of studies, most with limited sample sizes, reduces the robustness and generalizability of the conclusions. Fourth, publication bias cannot be excluded, as studies reporting null or inconsistent results may be underrepresented. Finally, only articles published in English and indexed in PubMed (with complementary Google searches) were included, potentially leading to the omission of relevant studies.

## 5. Conclusions

Isolation and quantification methods exhibit high variability, which strongly influences the overall quantity and quality of the exosomes obtained. Characteristics, including cargo composition, purity, and exosome integrity, must be considered when developing validated methods. Furthermore, emerging evidence suggests that PTBM preconditioning can increase exosome release from cells. In summary, rigorous standardization of protocols is essential to advance the scientific understanding and the clinical potential of exosome-based therapies.

## Figures and Tables

**Table 1:** Potential disadvantages of exosome isolation and quantification techniques, including the number, integrity, and specificity of the particles obtained.

Isolation Method	Disadvantages
- Ultracentrifugation (UC)	High speed can damage exosomes [42]. UC can cause mechanical damage, making it difficult to maintain the bioactivity and morphological integrity of exosomes [50].
- Ultrafiltration (UF)	Exosomes with moderate purity. Exosomes can be lost due to membrane damage [32], which can impair their ability to bind to and communicate with target cells [51].
- Precipitation	Co-precipitates with non-exosomal contaminants, including proteins and polymeric materials [43]. Common contaminants include residual proteins, lipids, and polymers from isolation methods, which can retain biological activity, leading to inaccurate conclusions about exosome-mediated processes [52].
- Immunoaffinity-based capture (IAC)	IAC can impair exosomes’ functional capacity [44].
- Microfluidic techniques	A less sensitive method with less pure isolated exosomes due to the complexity of biological samples, the size overlap between exosomes and other EVs, and the heterogeneity of exosomes [11,53].
**Quantification Method**	**Disadvantages**
- Nanoparticle tracking analysis (NTA)	The method also measures non-exosomal contaminants. NTA cannot distinguish EVs from other particles, such as lipoproteins [45].
- Flow cytometry (FCM)	Smaller vesicles are counted as single particles when the concentration of smaller vesicles is high in the sample and the scattering or fluorescence signal exceeds the detection limit [46].
- Tunable resistive pulse sensing (TRPS)	Insensitivity to smaller exosomes and smaller vesicles, which are counted as single particles [47].
- Dynamic light scattering (DLS)	This technique cannot analyze heterogeneous exosome populations [37].
- Surface plasmon resonance (SPR)	It has difficulty discriminating between specific and non-specific interactions and mass-sensitive and sensor area limitations [49].
- Single-particle interferometric reflectance imaging sensor (SP-IRIS)	It has a detection limit of 3.94 × 10^9^ for CD81 and 5.07 × 10^9^ particles/mL for CD63 [48].

**Table 2:** PubMed search strategy (1 January 1986–31 August 2025).

Concept	Search Terms Used
#1 Exosomes/EVs	“extracellular vesicles” OR “exosomes” OR related terms
#2 Sample type	“plasma” OR “serum” OR “platelet-rich plasma”
#3 Quantification	“concentration” OR “quantification”
#4 Particles	“particles” OR “particles/mL”
Combined search	#1 AND #2 AND #3 AND #4
Filters applied	“human”/excluded: “cancer” and “metastasis”

**Note:** Full search strings and translations are provided in the Supplementary Materials for reproducibility.

**Table 3:** Reported exosome concentrations in human plasma samples from healthy donors.

Author Year	Sample Type	N	Isolation Method	Quantification Method	Mean Concentration (Particles/mL)
Božič [65] 2019	Plasma	3	–	FCM	3.50 × 10^6^
Woud [68] 2022	Plasma (donor group)	36	NA	IFCM	1.26 × 10^8^
Yim [71] 2023	Plasma	6	–	nFCM	1.50 × 10^10^
Marić [72] 2024	Plasma	20	UC	NTA	3.95 × 10^10^
Wang [73] 2024	Plasma (control group)	–	EXODUS	NTA	4.82 × 10^10^
Kong [56] 2023	Plasma	5	No isolation	NTA	1.78 × 10^11^
		5	SEC-PF	NTA	9.02 × 10^10^ *
		5	DGUC-SEC	NTA	1.51 × 10^9^ *
Weber [69] 2023	Plasma (healthy donors)	10	SEC	NTA	1.15 × 10^9^
Lichá [70] 2023	Plasma without Dnase	4	IDGUC	NTA	8.80 × 10^8^
Zhang [62] 2022	Plasma	5	UC	NTA	2.38 × 10^10^
Mohammad [66] 2021	Plasma (controls)	9	Differential DUC	NTA	8.11 × 10^10^
Bendix [57] 2019	Plasma	–	All isolation methods combined	NTA	~2.00 × 10^10^
		–	All UC techniques	NTA	~5.00 × 10^10^
Wang [64] 2019	Plasma	–	HIC	NTA	1.15 × 10^9^
Soares [60] 2018	Plasma	–	TEI	NTA	8.30 × 10^8^
			ExoQ	NTA	7.80 × 10^8^
			ExoS	NTA	9.90 × 10^8^
Connolly [61] 2018	PFP	7	UC	NTA	1.01 × 10^11^
		7	MB	NTA	3.10 × 10^11^
		7	SPD	NTA	2.50 × 10^10^
Jamaly [63] 2018	PPP	10	HSC	NTA	1.80 × 10^10^
Mørk [59] 2016	PFP	20	SEC	NTA	4.50 × 10^8^ *
	PFP (ERI)	20	SEC	NTA	6.70 × 10^11^
Dragovic [58] 2011	PPP	–	UC	NTA	1.20 × 10^10^
Gelibter [67] 2022	Fresh plasma	3	SEC	TRPS	4.36 × 10^10^
Mørk [59] 2016	PFP	20	SEC	TRPS	9.05 × 10^8^
	PFP (ERI)	20	SEC	TRPS	1.70 × 10^9^

* Article results mean value. Abbreviations: DGUC, density gradient ultracentrifugation; ERI, estimated reference intervals; EXODUS, ultrafast-isolation system; FCM, flow cytometry; HIC, hydrophobic interaction chromatography; HSC, high-speed centrifugation; IDUG, iodixanol density gradient ultracentrifugation; IFCM, imaging flow cytometry; MB, magnetic bead; N, number of donors; NA, not applicable; nFCM, nanoflow cytometry; NTA, nanoparticle tracking analysis; PFP, platelet-free plasma; PPP, platelet-poor plasma; SEC, size exclusion chromatography; SEC-PF, SEC with protein fractionation; SPD, solid-phase depletion; TRPS, tunable sensitive pulse sensing; UC, ultracentrifugation.

**Table 4:** Reported exosome concentrations in human serum samples from healthy donors.

Author Year	Sample Type	N	Isolation Method	Quantification Method	Mean Concentration (Particles/mL)
Zhang [62] 2022	Serum	6	UC	NTA	4.23 × 10^10^
Malys [74] 2021	Serum	3	Exo-spin^TM^	NTA	4.98 × 10^10^
		3	DUC	NTA	9.90 × 10^10^
Soares [60] 2018	Serum	–	TEI	NTA	5.30 × 10^8^
			ExoQ	NTA	5.40 × 10^8^
			ExoS	NTA	6.90 × 10^8^
Helwa [75] 2017	Serum (ID)	6	miRCURY	NTA	1.62 × 10^11^ *
		6	TEIR	NTA	2.13 × 10^11^ *
		6	UC	NTA	8.45 × 10^8^ *
		6	ExoQuick	NTA	1.79 × 10^11^ *

* Article results mean value. Abbreviations: DUC, differential ultracentrifugation; ID, individual donors; N, number of donors; NTA, nanoparticle tracking analysis; mL, milliliters; TEI, total Exosome Isolation^TM^; TEIR, total Exosome Isolation reagent for serum; UC, ultracentrifugation.

**Table 5:** Data from articles with exosome quantification in human platelet-rich plasma samples from healthy donors.

Author Year	Sample Type	N	Isolation Method	Quantification Method	Mean Concentration (Particles/mL)
Cordero [77] 2025	Preconditioned autologous PRP (blue light 467 nm, 1.0 J/cm^2^, 37 °C)	3	UC	NTA	2.99 × 10^11^ *
	Preconditioned autologous PRP (blue light 467 nm, 2.0 J/cm^2^, 37 °C)	3	UC	NTA	2.53 × 10^11^ *
Rui [76] 2021	PRP with saline solution	3	UC	Nanoflow analysis	7.52 × 10^9^ *
	PRP activated with calcium gluconate	3	UC	Nanoflow analysis	5.85 × 10^10^ *
	PRP activated with thrombin	3	UC	Nanoflow analysis	4.87 × 10^10^ *
	PRP activated with thrombin and calcium gluconate	3	UC	Nanoflow analysis	7.16 × 10^10^ *

* Article results mean value. Abbreviations: N, number of donors; mL, milliliters; NTA, nanoparticle tracking analysis; PRP, platelet-rich plasma; PTBM, photothermal biomodulation; UC, ultracentrifugation.

## Data Availability

The raw data supporting the conclusions of this article will be made available by the corresponding author, E.S.-V.M. on request.

## Supplementary Materials

The following supporting information can be downloaded at: https://www.mdpi.com/article/10.3390/biomedicines13122970/s1, Table S1: PubMed strategy searches.

## Abbreviations

The following abbreviations are used in this manuscript:

DLS	Dynamic light scattering
EM	Electron Microscopy
FCM	Flow cytometry
IAC	Immunoaffinity-based capture
miRNA	MicroRNA
MSC-Exo	Mesenchymal stem cell-derived exosome
MV	Microvesicle
NTA	Nanoparticle tracking analysis
PBM	Photobiomodulation
PRP	Platelet-rich plasma
PTBM	Photothermal biomodulation
SD	Standard deviation
SEC	Size-exclusion chromatography
SOPs	Standardized operating procedures
SP-IRIS	Single-particle interferometric reflectance imaging sensor
SPR	Surface plasmon resonance
TRPS	Tunable resistive pulse sensing
UC	Ultracentrifugation
UF	Ultrafiltration
