# Supplementary material for: Human Blood Exosomes: Isolation and Characterization Methods, Variability, and the Need for Standardized Protocols—A Review
Source: Biomedicines. 2025 Dec 3;13(12):2970. doi: 10.3390/biomedicines13122970 (PMC12731227; doi:10.3390/biomedicines13122970)
Supplement: Supplementary file 1 [file biomedicines-13-02970-s001.zip › biomedicines-3945847-supplementary.pdf]

Supplemental Tables

Supplemental Table 1. PubMed strategy searches.

**Search #1:** ("extracellular vesicles") OR (exosomes) Filters: Humans, from 1986/1/1 - 2025/08/31 Sort by: Most Recent<br>("extracellular vesicles"[All Fields] OR "exosomal"[All Fields] OR "exosomes"[MeSH Terms] OR "exosomes"[All Fields] OR "exosome"[All Fields] OR "exosomic"[All Fields]) AND ((humans[Filter]) AND (1986/1/1:2025/08/31[pdat]))<br>**Translations**<br>exosomes: "exosomal"[All Fields] OR "exosomes"[MeSH Terms] OR "exosomes"[All Fields] OR "exosome"[All Fields] OR "exosomic"[All Fields]
**Search #2:** (((plasma) OR ("platelet-rich plasma")) NOT (cancer)) NOT (metastasis) Filters: Humans, from 1986/1/1 - 2025/08/31 Sort by: Most Recent<br>((("plasma"[MeSH Terms] OR "plasma"[All Fields] OR "plasmas"[All Fields] OR "plasma s"[All Fields] OR "platelet-rich plasma"[All Fields]) NOT ("cancer s"[All Fields] OR "cancerated"[All Fields] OR "canceration"[All Fields] OR "cancerization"[All Fields] OR "cancerized"[All Fields] OR "cancerous"[All Fields] OR "neoplasms"[MeSH Terms] OR "neoplasms"[All Fields] OR "cancer"[All Fields] OR "cancers"[All Fields])) NOT ("metastasi"[All Fields] OR "neoplasm metastasis"[MeSH Terms] OR ("neoplasm"[All Fields] AND "metastasis"[All Fields]) OR "neoplasm metastasis"[All Fields] OR "metastasis"[All Fields])) AND ((humans[Filter]) AND (1986/1/1: 2025/08/31[pdat]))<br>**Translations**<br>plasma: "plasma"[MeSH Terms] OR "plasma"[All Fields] OR "plasmas"[All Fields] OR "plasma's"[All Fields]<br>cancer: "cancer's"[All Fields] OR "cancerated"[All Fields] OR "canceration"[All Fields] OR "cancerization"[All Fields] OR "cancerized"[All Fields] OR "cancerous"[All Fields] OR "neoplasms"[MeSH Terms] OR "neoplasms"[All Fields] OR "cancer"[All Fields] OR "cancers"[All Fields]<br>metastasis: "metastasi"[All Fields] OR "neoplasm metastasis"[MeSH Terms] OR ("neoplasm"[All Fields] AND "metastasis"[All Fields]) OR "neoplasm metastasis"[All Fields] OR "metastasis"[All Fields]

Search #3: (concentration) OR (quantification) Filters: Humans, from 1986/1/1 - 2025/08/31 Sort by: Most Recent

("concentrate"[All Fields] OR "concentrated"[All Fields] OR "concentrates"[All Fields] OR "concentrating"[All Fields] OR "concentration"[All Fields] OR "concentrations"[All Fields] OR "quantification"[All Fields] OR "quantifications"[All Fields]) AND ((humans[Filter]) AND (1986/1/1: 2025/08/31[pdat]))

Translations

concentration: "concentrate"[All Fields] OR "concentrated"[All Fields] OR "concentrates"[All Fields] OR "concentrating"[All Fields] OR "concentration"[All Fields] OR "concentrations"[All Fields]

quantification: "quantification"[All Fields] OR "quantifications"[All Fields]

Search #4: (particles) OR (particles/mL) Filters: Humans, from 1986/1/1 - 2025/08/31 Sort by: Most Recent

("particle"[All Fields] OR "particle s"[All Fields] OR "particles"[All Fields] OR "particles ml"[All Fields]) AND ((humans[Filter]) AND (1986/1/1: 2025/08/31[pdat]))

Translations

particles: "particle"[All Fields] OR "particle's"[All Fields] OR "particles"[All Fields]

Search: #1 AND #2 AND #3 AND #4 Filters: Humans, from 1986/1/1 - 2025/08/31 Sort by: Most Recent

(("extracellular vesicles"[All Fields] OR ("exosomal"[All Fields] OR "exosomes"[MeSH Terms] OR "exosomes"[All Fields] OR "exosome"[All Fields] OR "exosomic"[All Fields])) AND ("humans"[MeSH Terms] AND 1986/01/01: 2025/08/31[Date - Publication]) AND (((("plasma"[MeSH Terms] OR "plasma"[All Fields] OR "plasmas"[All Fields] OR "plasma s"[All Fields] OR "platelet-rich plasma"[All Fields]) NOT ("cancer s"[All Fields] OR "cancerated"[All Fields] OR "canceration"[All Fields] OR "cancerization"[All Fields] OR "cancerized"[All Fields] OR "cancerous"[All Fields] OR "neoplasms"[MeSH Terms] OR "neoplasms"[All Fields] OR "cancer"[All Fields] OR "cancers"[All Fields])) NOT ("metastasi"[All Fields] OR "neoplasm metastasis"[MeSH Terms] OR ("neoplasm"[All Fields] AND "metastasis"[All Fields]) OR "neoplasm metastasis"[All Fields] OR "metastasis"[All Fields])) AND ("humans"[MeSH Terms] AND 1986/01/01: 2025/08/31[Date - Publication])) AND (("concentrate"[All Fields] OR "concentrated"[All Fields] OR "concentrates"[All Fields] OR "concentrating"[All Fields] OR "concentration"[All Fields] OR "concentrations"[All Fields] OR

("quantification"[All Fields] OR "quantifications"[All Fields])) AND ("humans"[MeSH Terms] AND 1986/01/01: 2025/08/31[Date - Publication])) AND (("particle"[All Fields] OR "particle s"[All Fields] OR "particles"[All Fields] OR "particles ml"[All Fields]) AND ("humans"[MeSH Terms] AND 1986/01/01:2025/08/31[Date - Publication]))) AND ((humans[Filter]) AND (1986/1/1:2025/08/31[pdat]))

Translations

exosomes: "exosomal"[All Fields] OR "exosomes"[MeSH Terms] OR "exosomes"[All Fields] OR "exosome"[All Fields] OR "exosomic"[All Fields]

humans[Filter]: humans[MH]

plasma: "plasma"[MeSH Terms] OR "plasma"[All Fields] OR "plasmas"[All Fields] OR "plasma's"[All Fields]

cancer: "cancer's"[All Fields] OR "cancerated"[All Fields] OR "canceration"[All Fields] OR "cancerization"[All Fields] OR "cancerized"[All Fields] OR "cancerous"[All Fields] OR "neoplasms"[MeSH Terms] OR "neoplasms"[All Fields] OR "cancer"[All Fields] OR "cancers"[All Fields]

metastasis: "metastasi"[All Fields] OR "neoplasm metastasis"[MeSH Terms] OR ("neoplasm"[All Fields] AND "metastasis"[All Fields]) OR "neoplasm metastasis"[All Fields] OR "metastasis"[All Fields]

humans[Filter]: humans[MH]

concentration: "concentrate"[All Fields] OR "concentrated"[All Fields] OR "concentrates"[All Fields] OR "concentrating"[All Fields] OR "concentration"[All Fields] OR "concentrations"[All Fields]

quantification: "quantification"[All Fields] OR "quantifications"[All Fields]

humans[Filter]: humans[MH]

particles: "particle"[All Fields] OR "particle's"[All Fields] OR "particles"[All Fields]

humans[Filter]: humans[MH]
